# Valence Band Structure of InAs_1−*x*_Bi_*x*_ and InSb_1−*x*_Bi_*x*_ Alloy Semiconductors Calculated Using Valence Band Anticrossing Model

**DOI:** 10.1155/2014/704830

**Published:** 2014-01-29

**Authors:** D. P. Samajdar, S. Dhar

**Affiliations:** ^1^Department of Electronics and Communication Engineering, Heritage Institute of Technology, Chowbaga Road, Anandapur, Kolkata 700107, India; ^2^Department of Electronic Science, University of Calcutta, 92, A. P. C. Road, Kolkata 700009, India

## Abstract

The valence band anticrossing model has been used to calculate the heavy/light hole and spin-orbit split-off energies in InAs_1−*x*_Bi_*x*_ and InSb_1−*x*_Bi_*x*_ alloy systems. It is found that both the heavy/light hole, and spin-orbit split *E*_+_ levels move upwards in energy with an increase in Bi content in the alloy, whereas the split *E*_−_ energy for the holes shows a reverse trend. The model is also used to calculate the reduction of band gap energy with an increase in Bi mole fraction. The calculated values of band gap variation agree well with the available experimental data.

## 1. Introduction

Bi-containing III-V semiconductors are receiving great amount of interest in the last few years owing to their potential application in optoelectronic devices operating in the near to long infrared wavelength regions with enhanced capability. These materials are obtained by incorporating a small amount of Bi in the host semiconductor resulting in a major reduction in the material band gap. A comprehensive review of the state of the art in these materials has been provided in a recently published monogram [1]. Alloys, such as, GaAsBi, InGaAsBi, GaSbBi, InAsBi, and InSbBi, are being investigated for various target applications. The narrow band gaps InSb_1−*x*_Bi_*x*_ and InAs_1−*x*_Bi_*x*_ have been mostly investigated for applications in infrared detectors operating in the 3–5 and 8–12 *μ*m spectral range [2] though the large miscibility gap and very low equilibrium solid solubility of Bi in the host semiconductors present certain difficulties in their growth [3]. Presence of Bi is also reported to reduce the dependence of the energy band gap of the alloys with temperature [4] which makes it possible to fabricate laser diodes with temperature insensitive emission wavelengths [5]. In case of the well-investigated dilute III-V nitride alloys, the interaction of the N related resonant state with the conduction band of the host semiconductor causes the reduction in band gap. However, in III-V bismide alloys, the band gap reduction occurs due to a restructuring of the valence band as a result of the interaction of the Bi impurity level with the same band [6]. This interaction produces a splitting of both the heavy hole and the light hole energy bands into *E*_+_ and *E*_−_ energy levels where the split *E*_+_ level moves up in energy resulting in the observed band gap reduction. The valence band anticrossing (VBAC) model [7] has been successfully used to explain the behavior of III-V bismide materials. In this work, we have used this model to calculate the valence band structure of the narrow band gap semiconductors InAs_1−*x*_Bi_*x*_ and InSb_1−*x*_Bi_*x*_.

## 2. Mathematical Model

A theoretical model was used earlier to describe the restructuring of valence bands in the bismuth containing III-V semiconductors using **k·p** formalism [7]. The interaction of the impurity bismuth atoms with the valence band of the corresponding host semiconductor was described by a 12 × 12 Hamiltonian which included 6 p-like states of the semiconductor lattice atom and the 6 localized p-like states of the added impurity atoms [8]. At the Γ point, where **k** = 0, the 12 × 12 matrix can be written as:

(1) H0=(H00000V000000L00000V000000L00000V000000H00000V000000S00000V000000S00000VV00000EBi000000V00000EBi000000V00000EBi000000V00000EBi000000V00000EBi−SO000000V00000EBi−SO).

Here *H*, *S*, and *V* are given as [7]

(2) H=L=ΔEVBMx,S=12(L+H)−Δ0−ΔESOx,V=CBix.

In the above equations, Δ*E*_VBM_ and Δ*E*_SO_, respectively, denote the difference in valence-band maximum and spin-orbit split-off band energies between the end point compounds and *x* is the mole fraction of bismuth incorporated into the semiconductor alloy. Δ_0_ gives the value of the split-off energy gap of the host semiconductor which for InAs, InSb, and GaSb are 0.39 eV, 0.81 eV, and 0.76 eV, respectively [9]. *V* is the matrix element describing the coupling between the host valence band and the Bi related impurity level and *C*_Bi_ is the coupling parameter which is used as a fitting parameter in our model [10]. *E*_Bi_ denotes the position of the heavy/light hole levels of the impurity atoms and *E*_Bi−SO_ gives the corresponding spin-orbit split-off level [7].

The solution of the 12 × 12 matrix *H*_0_ yields four distinct eigen values corresponding to the heavy/light hole *E*_+_ and *E*_−_ energy levels and the spin-orbit-split off energy levels *E*_SO+_ and *E*_SO−_. Hence the 12 × 12 Hamiltonian reduces to a 4 × 4 matrix given by

(3) $$\begin{array}{c} H_{R}=\left(\begin{array}{cccc} L/H & 0 & V & 0\\ 0 & S & 0 & V\\ V & 0 & E_{\text{Bi}} & 0\\ 0 & V & 0 & E_{\text{Bi}-\text{SO}} \end{array}\right). \end{array}$$

On solving the above matrix, we get the relations for the four distinct energy levels as explained by the VBAC model and are given as

(4) $$\begin{array}{c} E_{\text{HH/LH}\pm }=\frac{1}{2}\left(L+E_{\text{Bi}}\pm \sqrt{L^{2}-2LE_{\text{Bi}}+E_{\text{Bi}}^{2}+4V^{2}}\right),\\ E_{\text{SO}\pm }=\frac{1}{2}\left(S+E_{\text{Bi}-\text{SO}}\pm \sqrt{S^{2}-2SE_{\text{Bi}-\text{SO}}+E_{\text{Bi}-\text{SO}}^{2}+4V^{2}}\right). \end{array}$$

## 3. Valence Band Structure for InAs_1−*x*_Bi_*x*_

**Figure 1:**
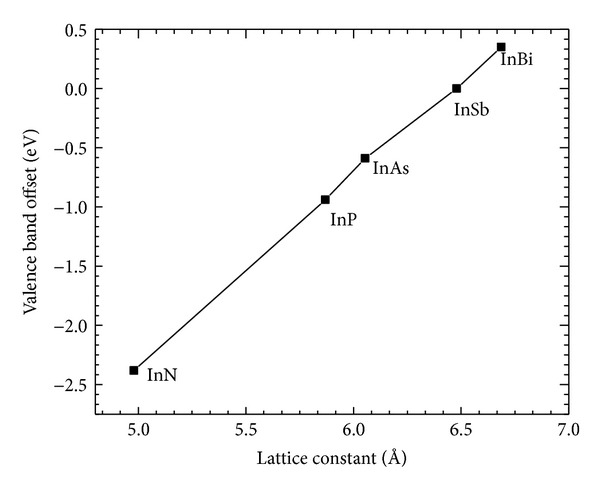
Plot of valence band offset versus lattice constant for In-containing III-V binaries. The values of VBO and lattice constants for these compounds are obtained from [9].

For VBAC calculations, we have considered Type I band alignment between InAs and InBi. The theoretically calculated band gap of InBi, as predicted by quantum dielectric theory by Barnett [11] is −1.62 eV. The valence band offset for InBi is found out to be 0.35 eV from Figure 1 (drawn by using the data from [9]) corresponding to its lattice constant of 6.686 Å [12]. The valence band offset Δ*E*_VBM_ between the end point compounds in InAs_1−*x*_Bi_*x*_ is obtained as 0.94 eV from an extrapolation of the variation of valence band offsets with lattice constants in Figure 1.

The value of the spin-orbit splitting energy for InBi is reported as 2.2 eV [12]. Hence the values of the valence band offset, conduction band offset, and spin-orbit split-off band offset for the end point compounds InAs and InBi are found out to be 0.94 eV, −1.03 eV, and −0.87 eV, respectively. Using the value of the atomic spin-orbit splitting energy for Bi of 1.5 eV [7], the position of the heavy/light hole levels for Bi, denoted by *E*_Bi_, is found to be located 0.4 eV below the valence band maximum (VBM) of InAs [5] and the location of corresponding spin-orbit split-off level *E*_Bi−SO_ is 1.9 eV below the VBM of InAs. The theoretical band gap of the ternary semiconductor is defined as the difference in energy between the VBAC calculated valence-band maximum and conduction band minimum obtained from the virtual crystal approximation (VCA) calculations, *E*_CB−VCA_, which can be written as [7]

(5) $$\begin{array}{c} E_{\text{CB}-\text{VCA}}=E_{g}-\Delta E_{\text{CBM}}x \end{array}$$

*E*_*g*_ is the band gap of InAs and Δ*E*_CBM_ = 1.03 eV is the conduction band edge offset between InAs and InBi. In InAs_1−*x*_Bi_*x*_, the reported band gap reduction is 55 meV per 1 at % of Bi in the host semiconductor [12]. Using this value, we have calculated the value of the fitting parameter *C*_Bi_ to be 1.26 eV.

**Figure 2:**
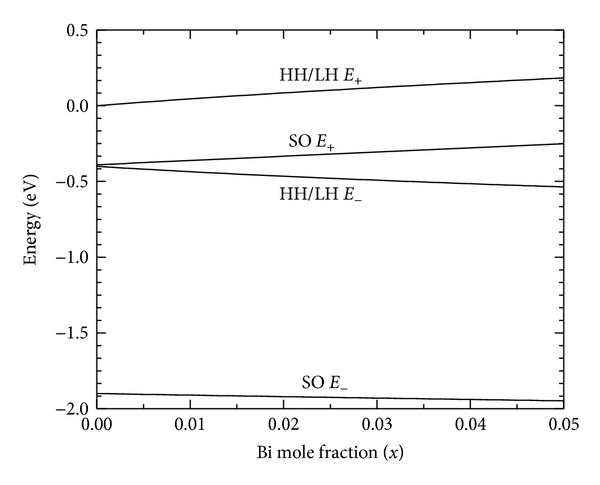
Position of the *E*_+_ and *E*_−_ related heavy/light hole and spin-orbit split-off bands as a function of Bi mole fraction for InAs_1−*x*_Bi_*x*_, calculated using VBAC model.

**Figure 3:**
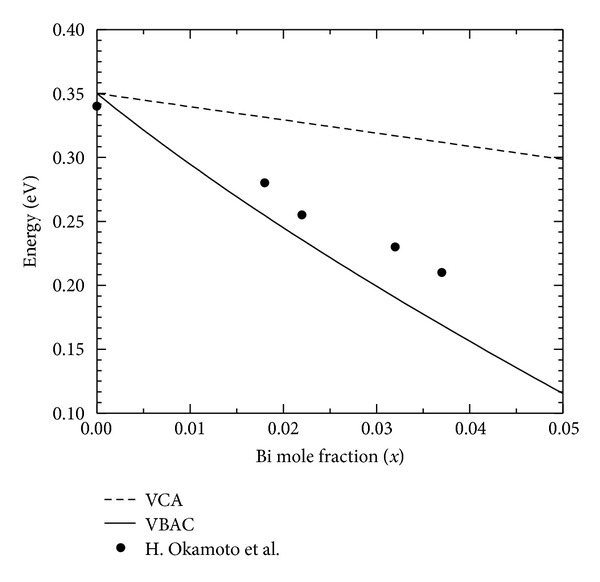
Theoretically calculated band gap of InAsBi as a function of Bi mole fraction using VCA and VBAC model. Experimental values of band gap are taken from [3].

Figure 2 shows the valence band structure of InAs_1−*x*_Bi_*x*_ as a function of Bi mole fraction *x*. The positions of the energy levels are calculated using (4). It can be noted from the figure that a repulsion exists between the *E*_+_ and *E*_−_ levels corresponding to the heavy hole/light hole bands and the spin-orbit split-off energy bands. Both the heavy hole and the light hole *E*_+_ levels are found to move up by about 45 meV for 1 at % Bi in the material with a corresponding downward movement of the *E*_−_ level. In the spin-orbit split-off band, the *E*_SO+_ level moves up by about 28 meV per at % Bi. The upward movement of the *E*_+_ level is mostly responsible for the band gap reduction in III-V bismides.  Figure 3 gives the variation of band gap as a function of Bi mole fraction calculated using relation (5) and VBAC. Satisfactory agreement of the theoretical prediction with available experimental values is shown in the figure.

## 4. Valence Band Structure for InSb_1−*x*_Bi_*x*_

The valence band anticrossing interaction in InSb_1−*x*_Bi_*x*_ can be modelled by considering a valence band offset of 0.59 eV between InSb and InAs as can be observed from Figure 1. Thus, assuming the constancy of the localized impurity levels relative to the vacuum level, the Bi related impurity level *E*_Bi_ is located 1.0 eV below the VBM of InSb, whereas the corresponding spin-orbit split-off band is located at a depth of 2.5 eV below the VBM of InSb. The values of Δ*E*_VBM_, Δ*E*_CBM_, and Δ*E*_SO_ are found in a way similar to that for InAs_1−*x*_Bi_*x*_ The value of Δ*E*_VBM_ obtained from Figure 1 is 0.35 eV. Using this value and the value of energy gap for InBi, the value of Δ*E*_CBM_ is found out to be −1.44 eV and the value for Δ*E*_SO_ is −1.04 eV. The value of the fitting parameter *C*_Bi_ is calculated as 0.33 eV using experimentally obtained band gap reduction of 19 meV per at % of Bi in InSbBi [13].

**Figure 4:**
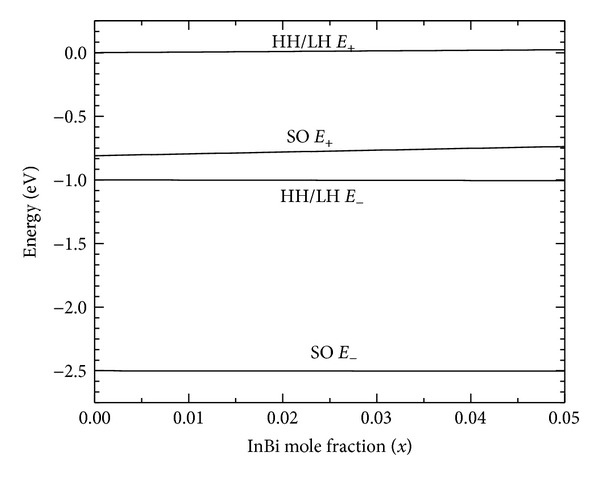
Calculated values of the *E*_+_ and *E*_−_ related heavy/light hole, and the spin-orbit split bands in InSb_1−*x*_Bi_*x*_, as a function of Bi mole fraction *x*.

**Figure 5:**
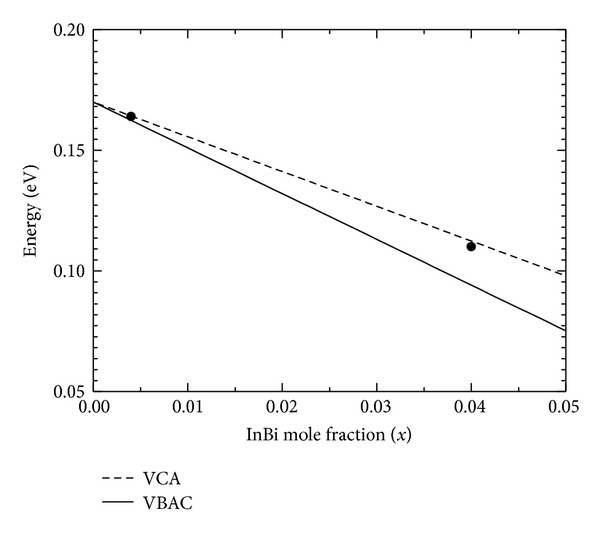
Band gap of InSbBi as a function of Bi mole fraction *x* calculated using VCA and VBAC model. The experimental points (solid dots) are from [14, 15].

Figure 4 presents the variation of the heavy/light hole and the spin-orbit split-off bands as a function of Bi mole fraction *x* for InSb_1−*x*_Bi_*x*_. Here the *E*_+_ level moves up by 4.6 meV for each at % Bi in the alloy, whereas the *E*_SO+_ level increases by 14.5 meV for the same amount of Bi. These values are smaller as compared to those for InAs_1−*x*_Bi_*x*_ due to the larger separation between the *E*_+_ and *E*_−_ levels and the SO_+_ and SO_−_ levels. This occurs due to the fact that the Bi related impurity level *E*_Bi_ and the spin-orbit split-off level *E*_Bi−SO_ are located at a greater depth from the VBM of InSb than in InAs which reduces the interaction between the coupled bands.  Figure 5 shows the reduction in band gap with the increase in Bi mole fraction calculated using VBAC and VCA model. A good agreement of this plot with experimental values is also presented in the same figure.

## 5. Conclusions

Valence band anticrossing model has been used to explore the valence band structures of Bi-containing alloys InAsBi and InSbBi. The theoretical results of band gap reduction agree fairly with the experimental data. The upward shift in the heavy/light hole *E*_+_ level and spin-orbit split-off level *E*_SO+_ is observed in both cases. This upward movement of the heavy hole/light hole *E*_+_ band is primarily responsible for the band gap reduction in these Bi-containing alloys.
